# Correlation of time trends of air pollutants, greenspaces and tracheal, bronchus and lung cancer incidence and mortality among the adults in United States

**DOI:** 10.3389/fonc.2024.1398679

**Published:** 2024-07-25

**Authors:** Jia Zhao, Ruihang Ren, Narasimha M. Beeraka, Mahesh PA, Nannan Xue, Pengfei Lu, Wenhua Bai, Zhihan Mao, Hemanth Vikram PR, Kirill V. Bulygin, Vladimir N. Nikolenko, Ruitai Fan, Junqi Liu

**Affiliations:** ^1^ Department of Thoracic Surgery, The First Affiliated Hospital of Zhengzhou University, Zhengzhou, China; ^2^ Department of Radiation Oncology & Cancer Center, The First Affiliated Hospital of Zhengzhou University, Zhengzhou, China; ^3^ Department of Pharmacology, Raghavendra Institute of Pharmaceutical Education and Research (RIPER), Andhra Pradesh, Ananthapuramu, India; ^4^ Department of Human Anatomy and Histology, I.M. Sechenov First Moscow State Medical University of the Ministry of Health of the Russian Federation (Sechenov University), Moscow, Russia; ^5^ Herman B. Wells Center for Pediatric Research, Department of Pediatrics, Indiana University School of Medicine, Indianapolis, IN, United States; ^6^ Department of Pulmonary Medicine, JSS Medical College, JSS Academy of Higher Education & Research (JSS AHER), Mysuru, Karnataka, India; ^7^ Genetic and Molecular Epidemiology Group, Spanish National Cancer Research Centre (CNIO) and CIBERONC, Madrid, Spain; ^8^ Cancer Center of the First Affiliated Hospital of Xinjiang Medical University, Urumqi, China; ^9^ Department of Pharmaceutical Chemistry, JSS College of Pharmacy, JSS Academy of Higher Education & Research (JSS AHER), Mysuru, Karnataka, India

**Keywords:** air pollutants, particulate matter, greenspace, TBL cancer, age groups

## Abstract

**Background:**

Tracheal, Bronchus, and Lung (TBL) cancer continues to represent the majority of cancer-related incidence and mortality in United States (U.S.). While air pollutants are considered essential risk factors, both global and national average concentrations of major harmful air pollutants have significantly decreased over the decades. Green space may have a beneficial effect on human health.

**Methods:**

We obtained data on national and state-level burden of TBL cancer, the annual average concentration of main air pollutants, and levels of green spaces in 2007, 2013, and 2019. According to generalized estimating equation (GEE), we examine the associations among incidence and mortality of TBL cancer, air pollutants, and greenspaces, represented by the Normalized Difference Vegetation Index (NDVI) in different age groups with models adjusted with meteorological, and socio-demographic. We observed additional effects of the interaction between the NDVI, Ozone, PM2.5, and other factors, which helped us to interpret and understand our results. Also, we collated states that witnessed net increments in forest coverage and conducted the same analysis separately.

**Results:**

In our analysis, the majority of associations between NDVI and air pollutants with TBL cancer remained significantly positive, particularly noticeable among individuals aged 20 to 54. However, our findings did not explore air pollution as a potential mediator between greenspace exposure and TBL cancer. While the associations of PM2.5 with TBL cancer remained positive, the other four pollutants showed positive but statistically insignificant associations. Our interaction analysis yielded that there were positive associations between NDVI and ozone, PM2.5, and tobacco use. Max NDVI acts as a protective factor along with high HDI. Additionally, PM2.5 and HDI also showed a negative association. In 18 states with more forest, NDVI acts as a protective factor along with higher health care coverage, better health status, and participation in physical activities.

**Conclusion:**

In the state-level of U.S., the effects of total greenspace with TBL cancer are mixed and could be modified by various socio-economic factors. PM2.5 has a direct correlation with TBL cancer and the effects can be influenced by underlying socioeconomic conditions.

## 1 Introduction

Tracheal, bronchus, and lung (TBL) cancer stands as one of the significant contributors to cancer-related fatalities in U.S. as of 2023. It is projected to constitute 21% of cancer-related deaths and 12% of cancer diagnoses. Despite its profound impact on public health, TBL cancer exhibits a comparatively low survival rate (1, 2). According to the Cancer Tomorrow report by GLOBOCAN, incident cases of TBL in U.S. are forecasted to surge by approximately 38% by 2040, with mortality cases expected to rise by about 45%. Furthermore, the majority of TBL cancer cases in U.S. occur in individuals aged 50 and above. However, roughly 20% of total TBL cancer deaths are not linked to tobacco use, potentially placing it as the eighth most prevalent cause of cancer-related mortality (1, 3, 4).

According to the International Agency for Research on Cancer (IARC), outdoor air pollution is classified as a Group 1 human carcinogen and significantly contributes to the burden of diseases, including lung cancer (5). Moreover, ambient PM2.5 air pollution has been identified as a contributing factor in nearly 14.1% of lung cancer deaths globally (6). The persistence of unhealthy air pollution levels for more than one-third of Americans underscores the need for continued research and public health interventions (1, 4, 7). The evolutionary and developmental mechanisms underlying TBL cancer remain complex. Exposure to air pollution may play a role in the progression of TBL cancer through the activation of signal transduction pathways, DNA damage, inflammation, metabolism, and epigenetic regulation (8). Recent studies have revealed a mechanistic relationship between air pollution and lung cancer, both in functional mouse models and clinical cohorts (9–11).

Based on the findings from seven European cohorts participating in the “Effects of Low-level Air Pollution: a Study in Europe” (ELAPSE), researchers have discovered a significant link between long-term exposure to PM2.5 and an increased risk of lung cancer, even at concentrations below the current European Union (EU) limit values. Similarly, the Adventist Health and Smog Study-2 (AHSMOG-2) cohort study also observed significant positive associations between incident lung cancer and PM2.5 exposure, particularly among individuals who have never smoked or are past smokers, at low concentrations (12, 13). Moreover, a case study in Canada suggests that never-smoking patients have indicated relationships with air pollution exposures, and chronic exposure to home air pollution is linked to an elevated risk of lung cancer in Nepal among never-smokers (14, 15). Besides, more than half of lung cancer patients in Taiwan are individuals who have never smoked. Elevated levels of PM2.5 can influence both the occurrence and the survival of patients with adenocarcinoma lung cancer (16, 17). Similarly, in Pennsylvania and California, air pollution could affect the survival rates of lung cancer patients following diagnosis (18, 19).

Greenspace was considered to provide health benefits and higher access to greenspace is positively correlated with longer life expectancy (20–22). Exposure to green spaces is inversely associated with overall and cause-specific mortality, indicating that it provides a protective role (23–25). For TBL cancer, green spaces may act as a protective role or work as an effective tool to improve air quality. Studies in France and Belgium have reported varying associations between exposure to greenspace, cancer incidence, and mortality. However, a retrospective cohort study in Taiwan revealed that increased exposure to green space may mitigate the harmful impacts of PM2.5 and reduce the risk of lung cancer (26–28). For other cancers, including breast and prostate cancer, there might be a protective relationship between exposure to greenspace and the development of malignancies (29).

Despite extensive studies on the health benefits of green spaces and the detrimental effects of air pollution, the specific interactions between green space, air pollution, and TBL cancer at the state level in the United States remain underexplored. This study addresses the following gaps: (1) Limited exploration of green space and cancer links: While existing research validates the general health benefits of green spaces, there is a lack of detailed exploration regarding their specific association with TBL cancer. This study aims to provide a more comprehensive understanding of how green spaces influence TBL cancer incidence and mortality. (2) Reconfirmation of PM2.5 impact: Although the link between PM2.5 air pollution and TBL cancer has been established, this study reconfirms this connection within the context of green space exposure, emphasizing the need to consider multiple environmental factors simultaneously. (3) Need for nuanced analysis: Current research often overlooks the complex interactions between green spaces, air pollution, lung cancer, and varying population demographics. This study highlights the necessity of a more nuanced and precise analysis that accounts for these interactions to better inform future research and public health policies.

By addressing these gaps, the study aims to enhance our understanding of the interplay between environmental factors and TBL cancer, ultimately providing valuable insights for the development of more effective health interventions and policies. Therefore, in this study, we conducted a comprehensive analysis to assess the interplay between green spaces, air pollutant levels, and the risk of tracheal, bronchus, and lung (TBL) cancer across different age groups (over 20 years, 20–54 years, and over 55 years) at the state level in U.S. Furthermore, we explored potential underlying mechanisms by investigating their associations with meteorological, sociodemographic, and socioeconomic factors.

## 2 Materials and methods

### 2.1 Study design and population

The contiguous U.S. spans approximately 9,834,633 square kilometers and is inhabited by nearly 330 million people. It comprises 50 states and the District of Columbia. Our analysis focused on 48 states along with the District of Columbia. The health system at the state level in U.S. holds significant authority. Alaska and Hawaii, along with overseas territories, were excluded from our research due to the non-applicability of study variables (30).

### 2.2 Data collection and measurements

#### 2.2.1 Outcome: TBL cancer incidence and mortality data

The Global Burden of Diseases (GBD) data visualization tool was developed by the Institute for Health metrics and Evaluation (IHME) in U.S (31). It provides statistics on the incidence and mortality rate of TBL cancer data by state, age, and year. Using the terminology defined in the GBD research, the state-wide incidence and mortality rate of TBL cancer were calculated (32). GBD is a reliable data source in U.S., compiling and summarizing data from various national databases. For individuals aged 20 and older, 20 to 54, and over 55, we estimated the incidence and mortality rate of TBL cancer for the 48 states and the District of Columbia, spanning three specific years: 2007, 2013, and 2019. We rounded the collected epidemiological data to integers for the convenience of subsequent research.

#### 2.2.2 Exposures and other variables

##### 2.2.2.1 Area greenness

In accordance with the US Environment Protection Agency (EPA), “greenspace” is any vegetated land, including gardens, lawns, forests, wetlands, and agricultural land (33). Using the NDVI, green space was estimated based on land surface reflectance, NDVI is a remote sense indicator that has been extensively utilized in epidemiological studies to evaluate the association between greenness and health. The following formula was used to obtain the NDVI proportions: NDVI = (NIR − R)/(NIR + R) (34). The MODIS images, composed of surface reflectance images updated every 16 days with a spatial resolution of 1000m per pixel, were primarily employed to assess the greenness on www.gisrs.cn (accessed on 30 April 2023). This website offers annual state-level estimates of the NDVI in U.S. An NDVI value of ‘0’ indicates the absence of vegetation, while values approaching ‘1’ indicate the highest level of greenness (35).

##### 2.2.2.2 Air pollution

Data sources from EPA were used to calculate the concentrations of main ambient air pollutants (36). The routine air quality monitoring stations in the U.S. gathered annual average concentrations of nitrogen dioxide (NO_2_), sulfur dioxide (SO_2_), ozone (O_3_), and particulate matter with diameters up to 2.5 µm (PM2.5) and 10 µm (PM10) for the years 2007, 2013, and 2019. We downloaded the average annual data of different time nodes for corresponding states and calculated the average values. The data were measured in various units, including parts per billion (ppb) and parts per million (ppm), and these were converted to micrograms per cubic meter (µg/m³).

##### 2.2.2.3 Weather and other variables

Data from the National Centers for Environmental Information (NCEI) and the Statewide Mapping and Climate at a Glance online sources were utilized to gather meteorological information for the states across U.S. for the years 2007, 2013, and 2019 (37). We selected a time scale of 12 months to obtain the yearly average temperature(°F) and annual precipitation(in inches). Data on PD (population density) and GDP (gross domestic product) for each state were compiled from the U.S. Bureau of Economic Analysis and the U.S. Census Bureau (38, 39). In assessing educational attainment, we used the rate of college completion as an indicator of higher education levels (40). State-level tobacco use age-adjusted prevalence was sourced from the BRFSS Prevalence & Trends Data portal (41). For the state-level HDI, we acquired the data from https://globaldatalab.org/shdi/, version v7.0 (42). Additionally, we collected potential socioeconomic factors, including healthcare coverage, health status, obesity rate, and participation in physical activities (43). We did not adjust the collected relevant factors as they met the criteria for our research. To explore the impact of different types of green spaces, We identified 18 states with increasing net forest coverage from 2000 to 2020 (44).

### 2.3 Statistical analysis

Utilizing state-level map charts and data visualization techniques, we depicted the variations in mean NDVI, TBL cancer incidence, and mortality across 48 states and Washington D.C. from 2007, 2013, to 2019. Two-dimensional multiple line graphs were employed to explore the relationship between TBL cancer data and mean NDVI (Y1 and Y2-axis) over the specified periods on the horizontal axis (X-axis), using R studio. The statistical software R, v.4.3.0, facilitated the plotting of multiple variables, utilizing gg-plot for bubble charts. The bubble plot, resembling a scatterplot, portrayed the average concentrations of pollutants and mean NDVI across the X-axis and Y-axis.

Initially, we assessed the correlations among air pollutants and between air pollutants and meteorological factors using Spearman’s correlations. Subsequently, the association between NDVI and TBL cancer incidence and mortality for each year was examined, controlling for individual air pollutants. A Poisson regression model was applied for each year, adjusting for meteorological parameters, GDP, and population density. Then, integrating the three-time points into a single model using a Generalized Estimating Equation (GEE) with a Poisson link, we estimated the association between NDVI (as the primary exposure variable) and TBL cancer incidence and mortality across different subgroups. Additionally, we investigated how NDVI and air pollution interacted with other relevant factors over the three time periods in the GEE model. All statistical analyses were two-sided, with effect estimates and 95% confidence intervals (CI) provided for associations with a p-value less than 0.05, indicating strong evidence of association. These statistical analyses were conducted using R Studio.

## 3 Results

**Figure 1:**
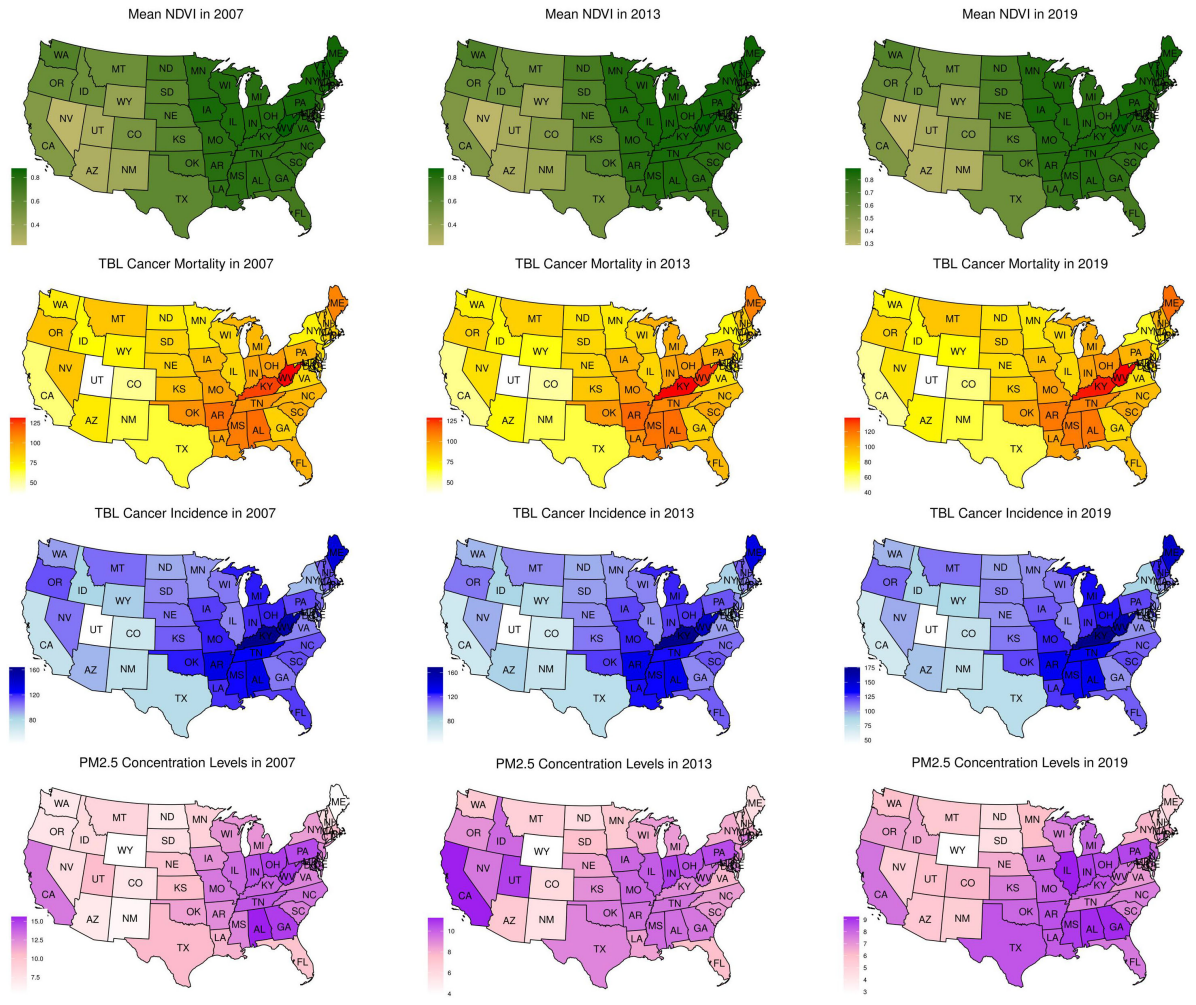
Distribution of mean NDVI, TBL cancer mortality, TBL cancer incidence, and mean PM2.5 concentration levels in ages over 20 years in the U.S. in 2007, 2013, and 2019. The normalized differential vegetation index (NDVI) values for 2007 (left), 2013 (middle), and 2019 (right), are shown on a spectrum of light green (least value) to dark green (highest value). Mean NDVI values of 0 to 0.2 are categorized as low, >0.2 to 4.0 as moderate, and >0.4 as high levels of greenspaces. Rates of TBL cancer incidence and mortality in ages over 55 years are shown on a spectrum of white (least value) to red (highest value) and dark blue (highest value).

Although TBL cancer incidence and mortality in over 20 years subgroup peaked in 2019 with relatively similar numbers (86.87 in 2007; 82.33 in 2013, and 88.69 in 2019), overall trends remained consistent and declined over the three time periods. The overall incidence and mortality rates of adult TBL cancer for individuals aged 20–54 years and over 55 years decreased across the three time periods (**Table 1**). The East South-Central region of U.S. showed higher rates of TBL cancer incidence and mortality among individuals over 55 years (**Figure 1**). The overall mean NDVI values in U.S. ranged from 0.70 in 2007 to 0.71 in 2013 to 0.72 in 2019 (**Figure 1**). The East and East Central regions of U.S. had higher mean NDVI values in 2007, 2013, and 2019. Conversely, the Western region, including states with high altitudes such as Arizona, Nevada, Utah, New Mexico, and Wyoming, exhibited comparatively lower NDVI values.

**Table 1:** Summary statistics for the incidence and mortality rates of Tracheal, Bronchus, and Lung (TBL) cancers, along with other socio-demographic factors in U.S. states for the years 2007, 2013, and 2019.

Measures	2007			2013			2019		
	Mean(SD)	Median(IQR)	Max,Min	Mean(SD)	Median(IQR)	Max,Min	Mean(SD)	Median(IQR)	Max,Min
TBL cancer mortality in 20+	86.87(±18.15)	85.78(98.74-74.84)	131.45,34.84	84.23(±19.00)	82.33(97.03-72.22)	128.65,34.96	89.92(±20.66)	88.69(103.82-77.74)	138.09,36.94
TBL cancer mortality in 20-54	12.21(±3.62)	11.51(14.64-10.03)	21.28,4.91	10.26(±3.37)	9.42(12.64-8.03)	18.56,3.98	8.75(±2.77)	8.21(10.42-6.98)	15.65,3.52
TBL cancer mortality in 55+	239.14(±40.34)	237.57(264.24-217.94)	339.60,123.74	212.49(±40.17)	205.08(238.10-188.54)	319.23,112.49	210.46(±40.99)	206.79(240.14-182.02)	318.14,11.03
TBL cancer incidence in 20+	106.01(±21.42)	108.19(119.39-93.46)	164.08,41.33	104.13(±22.80)	103.15(118.52-92.38)	168.88,41.52	110.40(±24.55)	110.31(127.21-97.35)	175.73,42.95
TBL cancer incidence in 20-54	17.02(±4.68)	16.32(20.20-13.93)	29.15,6.74	14.58(±4.53)	13.69(17.77-11.73)	28.14,5.54	12.36(±3.68)	11.47(14.71-10.06)	23.23,4.82
TBL cancer incidence in 55+	287.49(±46.89)	291.88(318.81-262.33)	438.97,144.11	259.34(±47.09)	259.58(288.92-228.42)	412.51,131.60	255.84(±47.21)	258.00(287.61-229.87)	403.67,127.51
Environmental factors									
MaxNDVI	0.90(±0.05)	0.92(0.93-0.89)	0.96,0.71	0.91(±0.05)	0.92(0.94-0.91)	0.98,0.73	0.92(±0.04)	0.93(0.94-0.91)	0.97,0.72
Mean NDVI	0.70(±0.17)	0.77(0.82-0.61)	0.88,0.23	0.71(±0.18)	0.80(0.83-0.60)	0.87,0.23	0.72(±0.16)	0.80(0.84-0.64)	0.89,0.29
Air pollutants									
PM2.5(μg/m³)	10.94(±2.81)	11.52(13.09-8.20)	15.61,5.31	8.45(±1.56)	8.75(9.60-7.31)	11.25,3.90	7.00(±1.47)	7.39(8.18-5.98)	9.25,2.85
PM10(μg/m³)	23.50(±6.64)	23.44(27.08-20.09)	52.51,10.99	19.53(±7.02)	18.17(21.27-14.89)	43.00,8.85	16.79(±5.28)	16.28(19.10-13.89)	38.33,7.18
SO2(μg/m³)	24.88(±14.07)	23.14(36.15-13.63)	75.86,3.21	10.42(±7.10)	8.54(14.64-5.07)	38.45,2.16	6.58(±6.07)	5.07(7.64-2.97)	34.39,0.68
NO2(μg/m³)	44.57(±20.22)	40.56(54.08-31.92)	103.13,8.49	34.85(±15.76)	31.41(42.35-25.42)	89.03,6.13	33.83(±14.26)	32.10(41.47-26.48)	75.65,6.04
OZONE(μg/m³)	86.09(±10.22)	87.52(94.83-78.52)	104.81,63.54	79.14(±6.80)	78.35(82.64-74.42)	96.29,64.14	78.28(±6.71)	77.11(83.80-73.03)	92.18,63.75
Meterorological variables									
Annual average temperature(degree Fahrenheit)	53.00(±7.79)	52.90(59.25-46.40)	71.5,40.8	51.59(±7.60)	50.30(57.00-45.25)	70.90,38.60	52.88(±8.74)	53.00(59.20-45.65)	73.50,38.30
Average precipitation(degree Inch)	34.06(±10.77)	36.31(42.24-28.62)	50.13,8.74	37.92(±15.37)	42.76(48.01-23.64)	63.50,7.28	41.82(±14.73)	46.48(49.88-31.52)	70.61,13.19
Socio-economic variables									
Population density	436.27(±1605.31)	108.80(242.25-49.95)	11280.00,5.90	395.75(±1404.40)	101.20(235.10-45.80)	9856.5,5.8	373.79(±1336.25)	88.70(220.55-41.40)	9370.60,5.10
GDP	316943.29(±373357.02)	189002.50(415680.35-90324.80)	2041192.20,26226.7	331896.06(±402848.75)	19169.80(436805-93593.80)	2179229.00,28681.50	382161.30(±488529.52)	219588.00(501016.95-97940.10)	2729225.80,29940.70

This table includes data on cancer incidence and mortality rates per 100,000 population. These factors are analyzed to understand the potential socio-demographic influences on cancer trends over time. Note: age groups (over 20 years, 20–54 years, and over 55 years).

There was a strong correlation between PM2.5 and SO_2_, while the other air pollutants showed weak to moderate relationships (**Supplementary Table S1**). PM2.5, PM10, NO_2_, and Ozone levels exhibited moderate and positive correlations with the annual average temperature. On the contrary, annual average precipitation was negatively and moderately correlated with PM10 and Ozone. Controlling for specific pollutants, NDVI was primarily associated with TBL cancer incidence and mortality in individuals over 20 years old across all three years (**Supplementary Table S2**). We also employed a unified model to evaluate all three years, adjusting for meteorological and socio-demographic factors. In this analysis, the associations between NDVI, air pollutants, and TBL cancer incidence and mortality remained largely positive.

The study found a significant association between the NDVI and the incidence of TBL cancer in individuals aged 20 and above. Specifically, for PM2.5, the association was quantified with a β coefficient of 0.713 (95% CI: 0.856, 1.140; p < 0.01). Similar significant associations were observed for other air pollutants: PM10 (β = 0.756; 95% CI: 0.590, 0.922; p < 0.001), SO2 (β = 0.692; 95% CI: 0.525, 0.859; p < 0.001), NO2 (β = 0.730; 95% CI: 0.557, 0.903; p < 0.001), and Ozone (β = 0.808; 95% CI: 0.626, 0.990; p < 0.001).

Similarly, NDVI was associated with TBL cancer mortality in the same age group. The β coefficient for PM2.5 was 0.664 (95% CI: 0.426, 0.902; p < 0.01). For other pollutants, the associations were: PM10 (β = 0.748; 95% CI: 0.579, 0.917; p < 0.001), SO2 (β = 0.669; 95% CI: 0.496, 0.842; p < 0.001), NO2 (β = 0.712; 95% CI: 0.540, 0.884; p < 0.001), and Ozone (β = 0.796; 95% CI: 0.610, 0.982; p < 0.001) (**Table 2**). Especially, more pronounced effects were observed among individuals aged 20 to 54 in terms of mortality (0.777 – 1.780) (**Table 2**). These findings were further supported by subgroup analyses, which demonstrated statistically significant correlations in both middle-aged individuals (20–54 years old) and older adults (55 years and above), reinforcing the overall relationship between NDVI, air pollution, and TBL cancer outcomes. The consistent significance across multiple pollutants and age groups underscores the robustness of the observed associations and suggests a broad impact of vegetation and air quality on TBL cancer incidence and mortality.

**Table 2:** Generalized estimation equation (GEE) result coefficients (β) (95% CI, lower, upper) of NDVI for association of TBL cancer incidence, mortality and air pollutants.

NDVI continuous	TBL cancer incidence in 20+	TBL cancer mortality in 20+	TBL cancer incidence in 20-54	TBL cancer mortality in 20-54	TBL cancer incidence in 55+	TBL cancer mortality in 55+
**Unadjusted**	0.726(0.499,0.953) **	0.673(0.440,0.906) **	0.281(0.068,0.494)	0.840(0.533,1.147) **	0.343(0.131,0.555)	0.281(0.068,0.494)
**Model 1**	0.998(0.856,1.140) ***	0.930(0.787,1.070) ***	0.558(0.431,0.684) ***	1.159(0.964,1.350) ***	0.636(0.511,0.762) ***	0.558(0.431,0.684) ***
**Model 2**	0.713(0.482,0.944) **	0.664(0.426,0.902) **	0.277(0.059,0.495)	0.777(0.467,1.087) *	0.334(0.118,0.550)	0.277(0.059,0.495)
**Model 3**	0.756(0.590,0.922) ***	0.748(0.579,0.917) ***	0.763(0.605,0.921) ***	1.830(1.579,2.081) ***	0.764(0.605,0.923) ***	0.763(0.605,0.921) ***
**Model 4**	0.692(0.525,0.859) ***	0.669(0.496,0.842) ***	0.559(0.401,0.717) ***	1.350(1.099,1.601) ***	0.578(0.424,0.732) ***	0.559(0.401,0.717) ***
**Model 5**	0.730(0.557,0.903) ***	0.712(0.540,0.884) ***	0.728(0.564,0.892) ***	1.780(1.530,2.030) ***	0.736(0.569,0.903) ***	0.728(0.564,0.892) ***
**Model 6**	0.808(0.626,0.990) ***	0.796(0.610,0.982) ***	0.755(0.585,0.925) ***	1.710(1.445,1.975) ***	0.765(0.595,0.935) ***	0.755(0.585,0.925) ***

Each of the study variables was used in the GEE with the Poisson link to explore the associations between the incidence and mortality of TBL cancer and NDVI as the primary exposure variable. The unadjusted variables are reported separately. In the multivariate analysis, Model 1: Each covariate and weather parameters; further adjusted for GDP, Mean temperature, Annual precipitation and population density with each of the air pollutants in Model 2: PM2.5; Model 3: PM10; Model 4: SO_2_; Model 5: NO_2_; Model 6: Ozone; “*” Indicates significant p-interaction values and is reported if p-int < 0.05; “**” Indicates significant p-interaction values and is reported if p-int < 0.01; “***” Indicates significant p-interaction values and is reported if p-int < 0.001. Note: age groups (over 20 years, 20–54 years, and over 55 years).

Furthermore, the associations of PM2.5 with TBL cancer remained significantly positive after adjusting for meteorological and socio-demographic variables, ranging from 0.012 to 0.080. Notably, PM2.5 was associated with TBL cancer mortality in individuals aged 20–54 years (β = 0.076; 95% CI =0.070, 0.082; p < 0.001) for PM10, along with SO2, NO2, and Ozone (β = 0.062; 95% CI = 0.053, 0.070; p < 0.001), (β = 0.079; 95% CI = 0.072, 0.085; p < 0.001), and (β = 0.080; 95% CI = 0.073, 0.087; p < 0.001), respectively (**Table 3**). Despite this, the associations for the other four pollutants were positive but not statistically significant (**Table 3**).

**Table 3:** Generalized estimation equation (GEE) result coefficients (β) (95% CI, lower, upper) of PM2.5 for association of TBL cancer incidence, mortality and air pollutants.

PM2.5	TBL cancer incidence in 20+	TBL cancer mortality in 20+	TBL cancer incidence in 20-54	TBL cancer mortality in 20-54	TBL cancer incidence in 55+	TBL cancer mortality in 55+
**Unadjusted**	0.015(0.009,0.021) *	0.016(0.010,0.023) **	0.033(0.028,0.038) ***	0.067(0.058,0.076) ***	0.031(0.026,0.036) ***	0.033(0.028,0.038) ***
**Model 1**	0.025(0.017,0.033) **	0.029(0.020,0.038) **	0.042(0.035,0.049) ***	0.078(0.065,0.091) ***	0.037(0.031,0.044) ***	0.042(0.035,0.049) ***
**Model 2**	0.017(0.013,0.022) ***	0.019(0.014,0.024) ***	0.035(0.031,0.038) ***	0.076(0.070,0.082) ***	0.033(0.029,0.036) ***	0.035(0.031,0.038) ***
**Model 3**	0.012(0.006,0.019) *	0.014(0.007,0.050)*	0.030(0.025,0.035) ***	0.062(0.053,0.070) ***	0.028(0.023,0.033) ***	0.030(0.025,0.035) ***
**Model 4**	0.015(0.010,0.020) **	0.017(0.012,0.021) ***	0.035(0.031,0.039) ***	0.079(0.072,0.085) ***	0.033(0.029,0.037) ***	0.035(0.031,0.039) ***
**Model 5**	0.022(0.017,0.026) ***	0.023(0.019,0.028) ***	0.039(0.035,0.043) ***	0.080(0.073,0.087) ***	0.037(0.033,0.041) ***	0.039(0.035,0.043) ***

Each of the study variables was used in the GEE with the Poisson link to explore the associations between the incidence and mortality of TBL cancer and PM2.5 as the primary exposure variable. The unadjusted variables are reported separately. In the multivariate analysis, Model 1: Each covariate and weather parameters; further adjusted for GDP, PD, Mean temperature, Annual precipitation and population density with each of the air pollutants in Model 2: PM10; Model 3: SO2; Model 4: NO2; Model 5: Ozone; “*” Indicates significant p-interaction values and is reported if p-int < 0.05; “**” Indicates significant p-interaction values and is reported if p-int < 0.01; “***” Indicates significant p-interaction values and is reported if p-int < 0.001. Note: age groups (over 20 years, 20–54 years, and over 55 years).

Our interaction analysis between NDVI and ozone revealed positive associations with all TBL cancer rates, while concentrations of other air pollutants showed no significant associations. Interaction between PM2.5 and HDI showed a negative association (β = -0.324; 95% CI = -0.177, -0.471; p < 0.05) (β = -0.351; 95% CI = -0.484, -0.219; p < 0.01) with TBL cancer incidence and mortality in individuals over 20 years old. The interaction between PM2.5 and educational level was negatively associated with TBL cancer rates in all subgroups except for TBL cancer incidence in individuals aged over 55. Our interaction analysis between tobacco use and PM2.5 concentration levels indicated a positive association (β = 2.710; 95% CI = 1.510, 3.910; p < 0.05) (β = 2.710; 95% CI = 1.640, 3.780; p < 0.05) with TBL cancer incidence and mortality in individuals over 20 years old. Additionally, the interaction between max NDVI and HDI was negatively associated with TBL cancer incidence in the individuals aged 20–54 (β = -25.053; 95% CI = -13.052, -37.054; p < 0.05), as well as TBL cancer mortality in individuals aged 20 to 54 and those aged over 55 (β = -64.227; 95% CI = -18.502, -82.794; p < 0.001) (β = -25.053; 95% CI = -13.052, -37.054; p < 0.05) (**Table 4**).

**Table 4:** GEE interaction analysis result (β) (95% CI, lower, upper) of related factors for association of TBL cancer incidence, mortality and air pollutants in different age groups.

	TBL cancer incidence in 20+	TBL cancer mortality in 20+	TBL cancer incidence in 20-54	TBL cancer mortality in 20-54	TBL cancer incidence in 55+	TBL cancer mortality in 55+
NDVI*OZONE	0.026(0.015,0.037)*	0.025(0.014,0.036)*	0.034(0.0260.042)***	0.039(0.024,0.055)*	0.034(0.025,0.042)***	0.034(0.026,0.042)*
PM2.5*HDI	-0.324(-0.177,-0.471)*	-0.351(-0.484,-0.219)**	-0.110(0.006,-0.226)	-0.283(-0.105,-0.461)	-0.081(0.062,-0.224)	-0.110(-0.006,0.226)
PM2.5*EDU	-0.229(-0.168,-0.289)***	-0.249(-0.193,-0.305)***	-0.108(-0.059,-0.157)*	-0.216(-0.134,-0.298)**	-0.087(-0.027,-0.147)	-0.108(-0.059,-0.157)*
PM2.5*SMOKE	2.710(1.510,3.910)*	2.710(1.640,3.780)*	0.751(-0.106,1.526)	-0.235(0.765,-1.235)	0.807(-0.333,1.947)	0.751(-0.065,1.567)
MaxNDVI*HDI	*2.067(-13.329,17.463)*	*-5.383(8.692,-19.458)*	-25.053(-13.052,-37.054)*	-64.227(-18.502,-82.794)***	*-24.50(12.00,37.00)*	-25.053(-13.052,-37.054)*

Models in first four rows were adjusted for GDP, population density, mean temperature, annual precipitation, and population density. In italicized MaxNDVI with HDI, model were adjusted for Mean temperature and Annual precipitation; “*” Indicates significant p-interaction values and is reported if p-int < 0.05; “**” Indicates significant p-interaction values and is reported if p-int < 0.01; “***” Indicates significant p-interaction values and is reported if p-int < 0.001. Note: age groups (over 20 years, 20–54 years, and over 55 years).

In the analysis of 18 states with increasing net forest coverage from 2000 to 2020, we observed additional effects between mean NDVI and health care coverage, health status, obesity rate, and participation in physical activities, separately. (**Supplementary Table S3**). Regarding health care coverage, better health status, and participation in physical activities, NDVI indicated a protective role. However, when interacting with high BMI individuals, NDVI was positively associated with TBL cancer rates.

**Figure 2:**
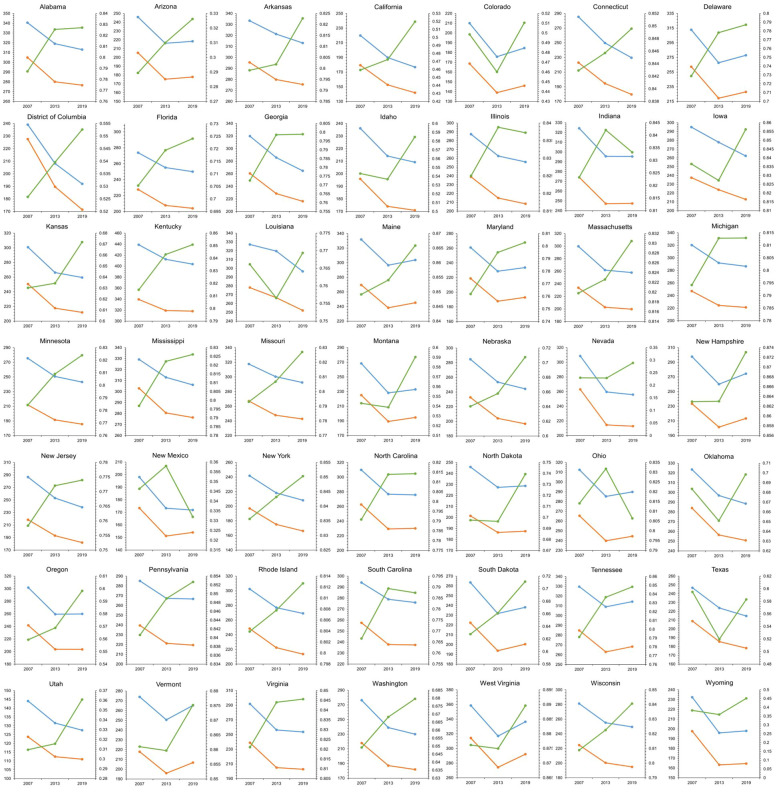
Data depicts the TBL cancer incidence and mortality (per 100,000 population) among individuals aged over 55 years, alongside the mean NDVI across 49 states in the United States for the years 2007, 2013, and 2019. Temporal trends are depicted by lines on the graph, with blue lines representing cancer incidence, orange lines representing cancer mortality, and green lines indicating the mean NDVI. The horizontal axis (X-axis) spans the three time periods of 2007, 2013, and 2019, while the Y1-axis on the left represents the cancer rate, and the Y2-axis on the right represents the mean NDVI.

The graph depicts the TBL cancer incidence and mortality rates (per 100,000 population) among individuals aged over 55 years and the mean NDVI across 49 states in the United States for the years 2007, 2013, and 2019 (**Figure 2**). The lines on the graph illustrate the temporal trends of the data series, with blue lines representing cancer incidence, orange lines representing cancer mortality, and green lines indicating the mean NDVI. The horizontal axis (X-axis) displays the three time periods (2007, 2013, and 2019), while the Y1-axis on the left side corresponds to the cancer rates, and the Y2-axis on the right side corresponds to the mean NDVI (**Figure 2**). Selected states in U.S. of our study: (1) Alabama, (2) Arizona, (3) Arkansas, (4) California, (5) Colorado, (6) Connecticut, (7) Delaware, (8) District of Columbia, (9) Florida, (10) Georgia, (11) Idaho, (12) Illinois, (13) Indiana, (14) Iowa, (15) Kansas, (16) Kentucky, (17) Louisiana, (18) Maine, (19) Maryland, (20) Massachusetts, (21) Michigan, (22) Minnesota, (23) Mississippi, (24) Missouri, (25) Montana, (26) Nebraska, (27) Nevada, (28) New Hampshire,(29) New Jersey, (30) New Mexico, (31) New York, (32) North Carolina, (33) North Dakota, (34) Ohio, (35) Oklahoma, (36) Oregon, (37) Pennsylvania, (38) Rhode Island, (39) South Carolina, (40) South Dakota, (41) Tennessee, (42) Texas, (43) Utah, (44) Vermont, (45) Virginia, (46) Washington, (47) West Virginia, (48) Wisconsin, and (49) Wyoming (**Figure 2**).

**Figure 3:**
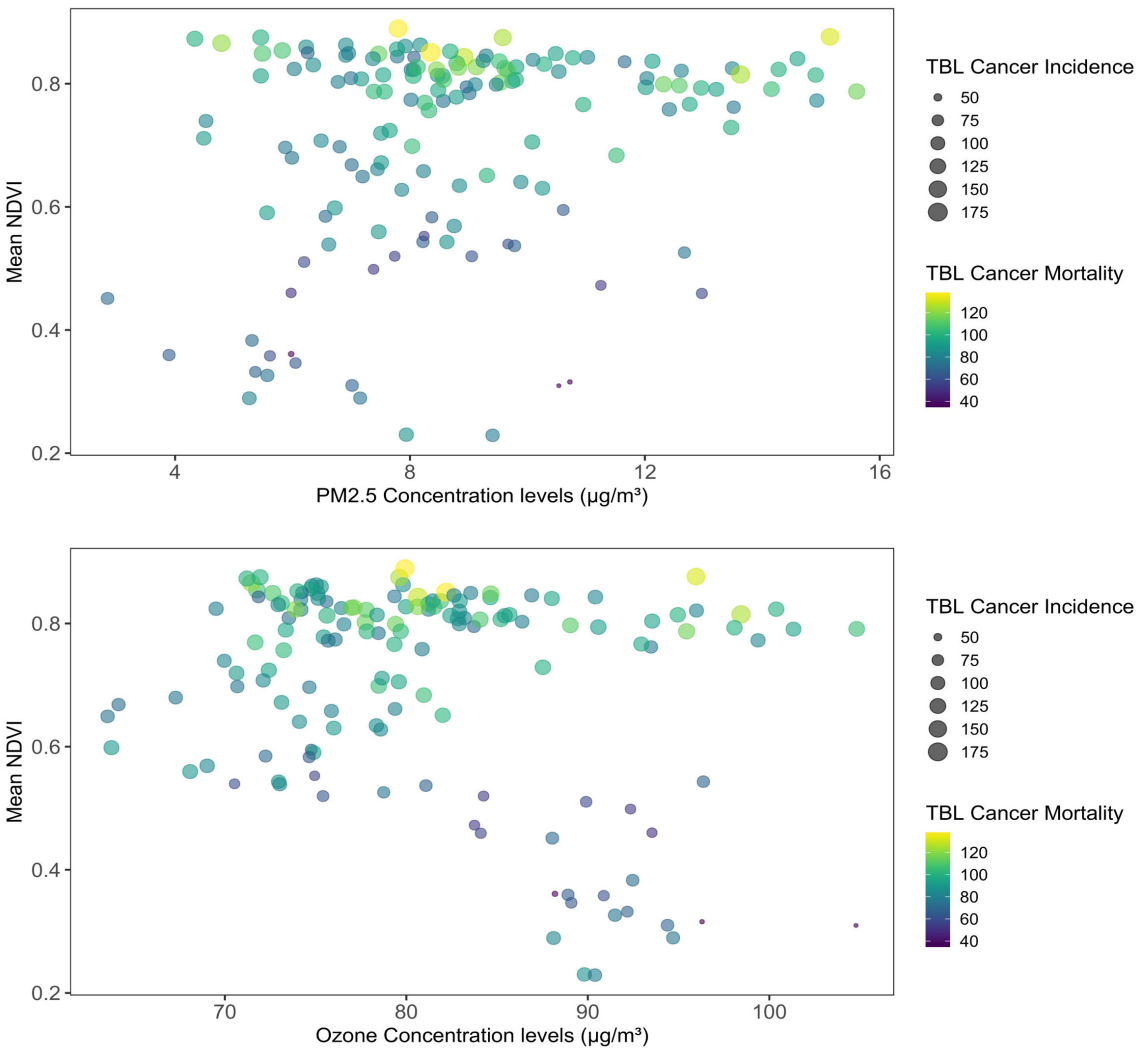
Mean NDVI and concentrations of PM2.5 and ozone across the states in the years 2007, 2013, 2019. Note: the left panel shows the association of the mean NDVI along the Y-axis and the PM2.5 or Ozone concentration levels on the X-axis. The size and colors of the bubbles measure the TBL cancer incidence and mortality of each state in the three time periods.

**Figure 3** presents the mean NDVI and concentrations of PM2.5 and Ozone across states for the years 2007, 2013, and 2019. In the left panel, the association between the mean NDVI (Y-axis) and the levels of PM2.5 or Ozone concentration (X-axis) is depicted. The size and color of the bubbles indicate the TBL cancer incidence and mortality for each state across the three time periods. Note: The data are represented as asthma prevalence (cases per 100,000 population), NDVI as the mean value, PM2.5 (particulate matter of diameter 2.5 µm or smaller), PM10 (particulate matter of diameter 10 µm or smaller), NO_2_ (nitrogen dioxide), SO_2_ (sulfur dioxide), O_3_ (ozone) in µg/m^3^, annual average temperature in degrees Fahrenheit, average annual precipitation in inches, GDP (gross domestic product), PD (population density) as persons/sqkm. Average values are presented as mean (± standard deviation), with minimum (Min) and maximum (Max) values, and percentile measures at 25th, 50th, 75th (**Figure 3**).

## 4 Discussion

In this research, we investigated the relationship between greenspace, air pollutant concentration, and TBL cancer across different age groups at the state level in U.S. Our findings uncovered a direct relationship between the average NDVI and PM2.5 levels with TBL cancer rates across all age groups, particularly notable in the 20 to 54 age range. This study stands as the first at the state level to identify such a connection. In our examination of interactions, we noted positive correlations between ozone and NDVI, as well as between PM2.5 and tobacco consumption. Conversely, we observed negative associations between PM2.5 concentration and educational attainment as well as HDI, and between maximum NDVI and HDI in the GEE models analyzing TBL cancer data.

Several studies have investigated the impact of greenspace on TBL cancer and other types of cancer. These studies have identified variability in the observed relationships, influenced by factors such as the type of greenspace, method of exposure measurement, individual attributes, and geographic location. For instance, studies in France and Spain found different results depending on the type of greenery. In France, spending more time near farmland was positively linked to a higher chance of getting breast cancer. Meanwhile, in Spain, it was linked to a higher risk of getting any type of cancer (26, 45). While exposure to neighborhood-level greenspace in Australia is speculated to be linked to higher risks of having skin cancer (46). In addition to a cross-sectional study conducted in Philadelphia, when green parcels as tiny as 1m² are included as greenspace, there is a positive correlation between the density of greenspace and both overall and cause-specific mortality (47). A recent meta-analysis compiled data from nine papers investigating the link between greenspace exposure and lung cancer, eventually, the combined findings suggested no significant association (29). Therefore, the relationship between green space and health appears to be nuanced, potentially varying based on geographical context, study methodologies, and the techniques used to assess exposure in the studies (48).

### 4.1 NDVI, Ozone, and TBL cancer

In our analysis of the interaction between maximum NDVI and HDI, we observed a protective effect of high greenspace in areas with high HDI. We theorized those individuals residing in high socioeconomic status areas, compared to those in low-income regions, exhibited lower rates of TBL lung cancer and stronger health preservation. This may be attributed to the likelihood that individuals with higher incomes are more inclined to benefit from the protective effects of greenspaces (49, 50). Additionally, we observed a positive correlation between NDVI and ozone levels with all TBL cancer rates. We considered the possibility that greenspaces could emit hydrocarbons, such as isoprene and terpenes, which serve as precursors to ozone. These biogenic hydrocarbons might contribute to the development of TBL cancer (51). According to research conducted in California, there was a moderate association observed between ozone levels and patients diagnosed with either squamous cell carcinoma or adenocarcinoma, which are two subtypes of lung cancer (19).

In the analysis of 18 states with increasing net forest coverage, we observed the NDVI exhibited a protective effect with higher healthcare coverage, improved health status, and engagement in physical activities (**Supplementary Table S3**). This indicates that the health impacts of different types of green spaces are diverse. States with net increases in forest coverage exhibit a more significant and positive effect on health promotion compared to states with unchanged or decreasing green spaces, demonstrating a better fit between the health effects and the presence of green spaces (52).

Our study did not account for other air pollution as a possible mediator between greenspace exposure and TBL cancer. However, it is hypothesized that certain trees and plants may release pollen, exacerbating allergies, while other aspects of urban vegetation may impede air circulation, leading to the accumulation of air pollutants (51). A study conducted in Los Angeles found higher levels of PM2.5 in areas adjacent to greenspaces compared to the parks or the broader region (49). This underscores the importance of considering potential unintended consequences when increasing green space in urban areas. For instance, it may create conditions favorable for the survival of infectious pathogens, potentially contributing to the spread of diseases (53). However, despite these considerations, the evidence regarding the association between access to green space and physical activity remains inconclusive (54).

### 4.2 PM2.5 and TBL cancer

With regard to our analysis, there is a significant positive relation between PM2.5 with TBL cancer mortality, which was similar to mortality attributable to risk factors of ambient particular pollution in the GBD compare visualization tool and one related research (55). As an avoidable cause of TBL cancer, attention to outdoor air pollution had perhaps been distracted away owing to the dominance of tobacco smoking. It is generally accepted that hazardous ambient emissions could be reduced to improve air quality, thus improving the morbidity and mortality of lung cancer (56). Interestingly, research on PM2.5 exposure in Europe, Japan, and Canada has uncovered several significant findings. Both long-term and short-term exposure to PM2.5 can contribute to lung cancer. Moreover, even exposure levels lower than the current EU limit values and possibly the WHO Air Quality Guidelines have been linked to lung cancer risk (12, 57, 58). In a population study conducted in Pennsylvania, researchers observed “U-shaped” dose-response curves, suggesting that both low and high exposures to PM10 can have a similar impact on lung cancer survival (18). In a cohort study in Canada, researchers applied a newly developed class of concentration-response models and observed sublinear associations between lung cancer incidence and PM2.5 (58). However, due to socioeconomic inequalities such as ethnicity, income level geography specificity, and so on, it is challenging to determine whether disparities in air pollution have been rising or falling in U.S (59). A study of U.S. veterans found that black individuals and those in socioeconomically deprived areas face a higher risk of PM2.5-related deaths from non-accidental and non-communicable causes, highlighting the impact of socioeconomic and racial factors (60).

In our interaction analysis, we found protective effects between PM2.5 and HDI, as well as educational level, respectively. Increased indoor PM2.5 concentrations were noted in rural U.S. households during winter, especially among those using wood stoves for heating. Additionally, urbanization overall has a notably positive impact on HDI (61, 62). Educational interventions have demonstrated efficacy in lowering indoor PM2.5 levels in specific subsets of households studied (63). It’s worth noting that air pollution can impact school performance, particularly in test scores. Air pollution does affect school performance, in particular test scores (64, 65). Regarding the combined effects of air pollution and smoking, we observed significant positive associations between PM2.5 and tobacco use. Hence, it is crucial to assess the risks of air pollution-related lung cancer alongside smoking risks. Remarkably, individuals with lung cancer who have never smoked showed significant associations with ambient air pollution exposure, compared to those who have ever smoked. Hence, it is vital to consider cumulative exposure to ambient air pollutants when assessing the risk of developing lung cancer, alongside accurately quantifying traditional risk factors like smoking. As smoking prevalence continues to decrease, the lung cancer risks associated with long-term current and previous ambient air quality may become relatively more important to public health (66).

### 4.3 Age Subgroup analysis

Our subgroup analyses based on age revealed some evidence of effect modification by selected individual characteristics. The TBL cancer mortality of 20–54 years subgroup was found to be strongest and positively associated with all the variables. Age appeared to modify the association between TBL cancer and exposure to PM2.5, with stronger impacts among young individuals compared to the elderly. This finding is consistent with a cohort study in Canada, which also indicated a tendency for stronger associations between lung cancer incidence with PM2.5 among younger adults (58). We attributed our findings to several factors distinguishing young adults from the elderly. For instance, they exhibit varying patterns of response to negative stimuli, with older adults showing different mobility performance compared to their younger counterparts. Additionally, young adults may not experience increased self-confidence due to their greater reliance on the Internet for cancer care. Notably, there are significant differences in immune profiles between adults and the elderly among colorectal cancer patients. A study conducted in U.S. revealed that the elderly group had notably higher levels of monocyte chemoattractant protein-1 (MCP-1) and lower levels of epidermal growth factor (EGF) compared to adults (67–71).

### 4.4 Limitations

Our study has a few limitations, one of which is the constrained accessibility of the EPA air quality monitoring network for measuring state-level air pollutant concentrations in specific cities. For greenspace, our measure of state-wide greenness was rather crude in that it lacked specification of the space type. Annual green space may be influenced by weather and geographical characteristics. Furthermore, contact with green space is indirect, complex, and multidimensional exposure. Green space including agriculture, lawns, forests, wetlands, and gardens, may contribute differently to public health. The risk of TBL cancer is influenced by numerous factors, and our findings regarding the association between green space exposure, air pollutants, socioeconomic factors, and TBL cancer should be interpreted cautiously. The benefits offered by green space may be overshadowed by other conditions and accompanying lifestyles. Additionally, our study’s findings may not apply to other regions globally due to differences in urban morphology, air quality improvement efforts, and car-oriented lifestyles, particularly in U.S. Unfortunately, we lacked information on other significant factors potentially related to cancer, which could confound our analyses. There is an urgent need for standardized methods of analysis when considering green space exposure and its various forms. Existing and future studies focusing on greenness in specific areas should be interpreted with caution (72). The characteristics such as access, biodiversity, facilities, and aesthetics would influence green spaces when exerting their health benefits to the public (73, 74). Future studies should aim to adjust for a comprehensive set of covariates, assess the quality of greenspace, and explore the association of greenspace exposure with different cancer subtypes (20, 23, 29, 75, 76).

## 5 Conclusion

In U.S., the distribution of greenspace and air pollution concentrations varies from east to west, with both factors being linked to TBL cancer along with distinct socioeconomic variables and other correlated risk factors. This study represents the first attempt to investigate the relationship between greenspace, air pollutants, socioeconomic factors, and TBL cancer at the state level in U.S. By correlating and contrasting with existing research on air pollution, greenspace, and various diseases, this study contributes to a better understanding of the association between the natural environment and health issues.

## Data availability statement

The original contributions presented in the study are included in the article/**Supplementary Material**. Further inquiries can be directed to the corresponding authors.

## Author contributions

JZ: Conceptualization, Writing – original draft, Writing – review & editing. RR: Conceptualization, Data curation, Methodology, Validation, Writing – original draft, Writing – review & editing. NB: Conceptualization, Data curation, Formal analysis, Writing – review & editing. MP: Conceptualization, Writing – review & editing. NX: Conceptualization, Writing – original draft, Writing – review & editing. PL: Conceptualization, Data curation, Writing – original draft, Writing – review & editing. WB: Conceptualization, Data curation, Writing – original draft, Writing – review & editing. ZM: Conceptualization, Data curation, Writing – review & editing, Writing – original draft. HP: Conceptualization, Data curation, Writing – original draft, Writing – review & editing. KV: Formal analysis, Writing – review & editing. VN: Writing – review & editing. RF: Conceptualization, Writing – review & editing. JL: Writing – original draft, Writing – review & editing.
