# Supplementary material for: Correlation of time trends of air pollutants, greenspaces and tracheal, bronchus and lung cancer incidence and mortality among the adults in United States
Source: Front Oncol. 2024 Jul 25;14:1398679. doi: 10.3389/fonc.2024.1398679 (PMC11306054; doi:10.3389/fonc.2024.1398679)
Supplement: Supplementary file 1 [file Table_1.pdf]

Table S1. Correlations and p-values between the air pollutants and meteorological variables

	**PM2.5**	**PM10**	**SO2**	**NO2**	**O3**	**Mean temperature**	**Precipitation**
PM2.5	1						
PM10	**0.4887**	1					
	0.000						
SO2	**0.6038**	**0.4244**	1				
	0.000	0.000					
NO2	**0.4716**	**0.3965**	**0.3965**	1			
	0.000	0.000	0.000				
O3	**0.3582**	**0.3773**	**0.4468**	**0.3368**	1		
	0.000	0.000	0.000	0.000			
Mean temperature	**0.4369**	**0.3039**	0.1269	**0.2214**	**0.2438**	1	
	0.000	0.000	0.1258	0.007	0.003		
Precipitation	0.0940	**-0.4121**	-0.1406	-0.1090	**-0.2938**	**0.3163**	1
	0.258	0.000	0.089	0.189	0.000	0.000	

Note: Data describes the Correlation matrix for the air pollutants and the meteorological factors. The corresponding p values are reported below each correlation co-efficient values. Significant p values that is $p<0.05$ are made bold. The variables are continuous represent PM2.5, particulate matter of diameter 2.5µm or less; PM10, particulate matter of diameter 10µm or less; NO2 , nitrogen dioxide; SO2 , sulfur dioxide; and, O3, ozone measured as µg/m3 ; The annual average temperature in degrees Fahrenheit) and annual precipitation (in inches).

Table S2. The estimates for TBL cancer incident rates and NDVI with each of the air pollutants concentrations for the year 2007, 2013, and 2019.

	**TBL Cancer Incident Rates in 20+ (95%CI)**			**TBL Cancer Incident Rates in 20-54 (95%CI)**			**TBL Cancer Incident Rates in 55+ (95%CI)**		
	2007	2013	2019	2007	2013	2019	2007	2013	2019
**NDVI continuous**									
**unadjusted**	**0.5886(0.4092,0.768)**	**1.216(0.9801,1.4519)**	**0.5969(0.3958,0.798)**	**0.5093(0.3904,0.6282)**	**1.046(0.8814,1.2106)**	0.2156(0.0713,0.3599)	**0.4754(0.367,0.5838)**	**0.9949(0.8464,1.1434)**	**0.3302(0.1987,0.4617)**
**PM2.5**	0.3652(0.1253,0.6051)	**1.182(0.9164,1.4476)**	**0.5837(0.3803,0.7871)**	0.1868(0.0283,0.3453)	**0.817(0.6348,0.9992)**	0.1635(0.0174,0.3096)	0.2141(0.0697,0.3585)	**0.8344(0.669,0.9998)**	**0.2893(0.1562,0.4224)**
**PM10**	**0.6033(0.4228,0.7838)**	**1.216(0.9801,1.4519)**	**0.5976(0.3966,0.7986)**	**0.5177(0.3981,0.6373)**	**1.046(0.8814,1.2106)**	0.218(0.0738,0.3622)	**0.4904(0.3814,0.5994)**	**0.9948(0.8463,1.1433)**	**0.3322(0.2008,0.4636)**
**SO2**	**0.5111(0.324,0.6982)**	**1.121(0.878,1.364)**	**0.602(0.4007,0.8033)**	**0.446(0.3223,0.5697)**	**0.9619(0.7926,1.1312)**	0.2246(0.0801,0.3691)	**0.425(0.3122,0.5378)**	**0.9084(0.7557,1.0611)**	**0.3375(0.2058,0.4692)**
**NO2**	**0.5696(0.3881,0.7511)**	**1.165(0.9173,1.4127)**	**0.5787(0.3766,0.7808)**	**0.4946(0.3745,0.6147)**	**0.9753(0.8029,1.1477)**	0.1912(0.0463,0.3361)	**0.4644(0.355,0.5738)**	**0.9421(0.7866,1.0976)**	**0.3023(0.1702,0.4344)**
**O3**	**0.6136(0.4189,0.8083)**	**1.201(0.9642,1.4378)**	**0.5688(0.3659,0.7717)**	**0.5084(0.3796,0.6372)**	**1.037(0.872,1.202)**	0.2029(0.0572,0.3486)	**0.4653(0.348,0.5826)**	**0.9845(0.8356,1.1334)**	**0.3107(0.1779,0.4435)**

Table S3. The estimates for TBL cancer death rates and NDVI with each of the air pollutants concentrations for the year 2007, 2013, and 2019.

	**TBL Cancer Death Rates in 20+ (95%CI)**			**TBL Cancer Death Rates in 20-54 (95%CI)**			**TBL Cancer Death Rates in 55+ (95%CI)**		
	2007	2013	2019	2007	2013	2019	2007	2013	2019
**NDVI** continuous									
**unadjusted**	**0.6289(0.4305,0.8273)**	**1.295(1.0317,1.5583)**	**0.4974(0.2757,0.7191)**	**1.085(0.5348,1.6352)**	**1.783(0.9809,2.5851)**	0.9042(0.1638,1.6446)	**0.5093(0.3904,0.6282)**	**1.046(0.8814,1.2106)**	0.2156(0.0713,0.3599)
**PM2.5**	0.3416(0.0763,0.6069)	**1.185(0.8903,1.4797)**	**0.4732(0.2488,0.6976)**	0.3807(-0.3657,1.1271)	1.633(0.7262,2.5398)	0.8576(0.1088,1.6064)	0.1868(0.0283,0.3453)	**0.817(0.6348,0.9992)**	0.1635(0.1135,0.2135)
**PM10**	**0.6395(0.4399,0.8391)**	**1.295(1.0317,1.5583)**	**0.4984(0.2768,0.72)**	**1.108(0.5539,1.6621)**	**1.783(0.9807,2.5853)**	0.906(0.166,1.646)	**0.5177(0.3981,0.6373)**	**1.046(0.8814,1.2106)**	0.218(0.0738,0.3622)
**SO2**	**0.5354(0.3288,0.742)**	**1.195(0.9237,1.4663)**	**0.5048(0.2827,0.7269)**	0.8721(0.2987,1.4455)	1.543(0.7145,2.3715)	0.9177(0.1757,1.6597)	**0.446(0.3223,0.5697)**	**0.9619(0.7926,1.1312)**	0.2246(0.0801,0.3691)
**NO2**	**0.6035(0.4028,0.8042)**	**1.215(0.9384,1.4916)**	**0.4809(0.2579,0.7039)**	1.014(0.455,1.573)	**1.705(0.8572,2.5528)**	0.8911(0.1434,1.6388)	**0.4946(0.3745,0.6147)**	**0.9753(0.8029,1.1477)**	0.1912(0.0463,0.3361)
**O3**	**0.6652(0.4499,0.8805)**	**1.281(1.0167,1.5453)**	**0.476(0.2523,0.6997)**	0.9288(0.3196,1.538)	**1.783(0.981,2.585)**	0.9194(0.1763,1.6625)	**0.5084(0.3796,0.6372)**	**1.037(0.872,1.202)**	0.2029(0.0572,0.3486)

Note: The model estimated the association between continuous NDVI and TBL cancer incident and death rates for 2007, 2013 and 2019, adjusting for each air pollutant PM2.5 , PM10 , NO2 , SO2 and O3 concentration. The TBL cancer incident rates, death rates and 95% confidence intervals (95% CI) are shown. NDVI: normalized difference vegetation index. Significant p values that is $p<0.05$ are made bold. All models are adjusted for annual average temperature, annual precipitation, gross domestic product, and population density.

Table S3. GEE interaction analysis between socioeconomic factors and TBL cancer incidence and mortality with NDVI among different age groups in 18 states with net increase in forest coverage.

	TBL cancer incidence in 20+	TBL cancer mortality in 20+	TBL cancer incidence in 20-54	TBL cancer mortality in 20-54	TBL cancer incidence in 55+	TBL cancer mortality in 55+
**MeanNDVI*Health Care Coverage**	-4.002(-3.812,11.816)	-5.701(1.564,-12.966)	-16.866(-10.061,-23.671)*	-17.851(-6.824,-28.878)	-14.579(-6.886,-22.272)	-16.866(-10.061,-23.671)*
**MeanNDVI*Health Status(Good or Better Health)**	-15.096(-9.013,-21.179)*	-16.456(-11.903,-21.009)***	-26.135(-21.473,-30.797)***	-28.107(-14.952,-41.262)*	-24.062(-17.652,-30.472)***	-26.135(-21.473,-30.979)***
**MeanNDVI*Obese (BMI 30.0 - 99.8)**	10.581(5.502,15.660)*	10.827(6.532,15.122)*	18.782(13.559,24.005)***	25.169(17.789,32.549)***	18.059(12.285,23.833)**	18.782(13.559,24.005)***
**MeanNDVI*Exercise(yes)**	-0.136(-0.061,-0.211)	-0.135(-0.075,-0.195)*	-0.258(-0.210,-0.306)***	-0.276(-0.156,-0.396)*	-0.250(-0.187,-0.313)***	-0.258(-0.210,-0.306)***

GEE interaction analysis result(β) (95% CI, lower, upper) of related factors for association of TBL cancer incidence, mortality. Note: Models were adjusted for GDP, population density, mean temperature, annual precipitation, and population density; "*" Indicates significant p-interaction values and is reported if p-int < 0.05; "**" Indicates significant p-interaction values and is reported if p-int < 0.01; "***" Indicates significant p-interaction values and is reported if p-int < 0.001
